# A parametric additive hazard model for time-to-event analysis

**DOI:** 10.1186/s12874-024-02180-y

**Published:** 2024-02-24

**Authors:** Dina Voeltz, Annika Hoyer, Amelie Forkel, Anke Schwandt, Oliver Kuß

**Affiliations:** 1 Biostatistics and Medical Biometry, Medical School OWL, Bielefeld University, Universitätsstr. 25, Bielefeld, 33615 Germany; 2 Department of Statistics, Ludwig-Maximilians-University München, München, Germany; 3 Nuvisan GmbH, Neu-Ulm, Germany; 4 Institute for Biometrics and Epidemiology, German Diabetes Center, Leibniz Center for Diabetes Research at Heinrich Heine University Düsseldorf, Düsseldorf, Germany

**Keywords:** Additive hazard, Parametric modeling, Survival analysis, Time-to-event model

## Abstract

**Background:**

In recent years, the use of non- and semi-parametric models which estimate hazard ratios for analysing time-to-event outcomes is continuously criticized in terms of interpretation, technical implementation, and flexibility. Hazard ratios in particular are critically discussed for their misleading interpretation as relative risks and their non-collapsibility. Additive hazard models do not have these drawbacks but are rarely used because they assume a non- or semi-parametric additive hazard which renders computation and interpretation complicated.

**Methods:**

As a remedy, we propose a new parametric additive hazard model that allows results to be reported on the original time rather than on the hazard scale. Being an essentially parametric model, survival, hazard and probability density functions are directly available. Parameter estimation is straightforward by maximizing the log-likelihood function.

**Results:**

Applying the model to different parametric distributions in a simulation study and in an exemplary application using data from a study investigating medical care to lung cancer patients, we show that the approach works well in practice.

**Conclusions:**

Our proposed parametric additive hazard model can serve as a powerful tool to analyze time-to-event outcomes due to its simple interpretation, flexibility and facilitated parameter estimation.

**Supplementary Information:**

The online version contains supplementary material available at 10.1186/s12874-024-02180-y.

## Background

Regression models for time-to-event outcomes are preferentially fitted by the proportional hazard model [9]. This is surprising because the hazard ratio, which is the generic effect estimate of that model, has been repeatedly criticized in recent years. Most relevant points of concern were that the hazard ratio (*i*) is interpretable only when mistaken as a relative risk [25], (*ii*) has a built-in selection or left truncation bias even in randomized trials [2, 14, 24], and (*iii*) is non-collapsible [12], meaning that adjusting for a covariate that is associated with the event will in general change the hazard ratio, even if this covariate is not associated with the exposure. To conquer these shortcomings, parametric survival models have been recommended as they are simpler, more informative, more robust, and have hazard, survival and probability density function directly available [18, 21].

A hazard-based model that does not suffer from the above mentioned problems is the additive hazard model introduced by Aalen [1]. Results from additive hazard models can be translated to a relative survival scale which is, for example, routinely used in cancer epidemiology when a cohort of cancer patients is compared to the general population in terms of expected survival [11]. Moreover, recent works showed that parameters of the additive hazard model do not suffer from non-collapsibility [19], at least when defined in continuous time [23]. However, the Aalen model is rarely used in applied research because it assumes a non-parametric hazard as well as time-dependent covariates. Of course, these properties give large flexibility in modeling, but also complicate parameter estimation considerably. In addition, Bradburn et al. [4] state that “[...] the model coefficients are not easy to understand, and as they change repeatedly over time, can offer no single quantifiable effect size”. As a partial solution for that problem, Lin et al. [17] proposed an additive hazard model for (constant,) time-independent covariates, but still use a semi-parametric hazard which may require lengthy computations for some statistical standard software [22].

As a remedy, in this article we combine the additive hazard model with time-independent (, i.e., constant) covariates and a parametric assumption for the baseline hazard. This will result in a number of advantages in terms of interpretation, possible model extensions, and also enables parameter estimation using every software that allows maximizing a hand-coded likelihood function. In the Methods section, we proceed by introducing the formal notation and deriving the model’s equations. Then, we report the settings of our simulation study. We illustrate the model using a study which investigated provision of medical care to lung cancer patients in the eastern part of Germany [3] and present all results in Results section. Finally, we conclude with a discussion (Discussion section).

## Methods

### Parametric additive hazard model

We start with the assumption that the hazard $h_{x}(t)$ with covariate vector *x* at time *t* can be expressed as

1 $$\begin{aligned} {h_{x}(t) = h_{0,\theta }(t)} + x\beta , \end{aligned}$$

with a parametric baseline hazard function $h_{0,\theta }(t)$ which is independent of the covariates. The parameter $\theta$ denotes the distribution parameters, which differ in terms of number and commonly used notation depending on the choice of the baseline distribution. The parameters $\beta$ are regression coefficients that measure the additive impact of covariates.

Using the well-known relations between hazard, probability density function (pdf) (*f*(*t*)) and survival function *S*(*t*), the corresponding pdf of model (1), $f_{x}(t)$, can be expressed in terms of the baseline pdf $f_0(t)$, the baseline survival function $S_{0, \theta }(t)$, and the covariates by

2 $$\begin{aligned} {f_{x}(t) = \frac{f_0(t) + x\beta S_{0, \theta }(t)}{\exp (t x\beta )}}. \end{aligned}$$

The complete derivation of the additive hazard model equation in terms of the pdf can be found in Additional file 1.

The corresponding survival function $S_{x}(t)$ is given by

3 $$\begin{aligned} {S_{x}(t) = \frac{S_{0, \theta }(t)}{\exp (t x_i\beta )} = 1 - F_{x}(t)}, \end{aligned}$$

with $F_{x}(t)$ denoting the cumulative distribution function (cdf).

Using an additive hazard regression model allows to estimate relative survival instead of hazard ratios [27]. Contrarily to hazard ratios, which quantify the average or constant effect of covariates on the hazard function, relative survival measures the cumulative effect of covariates on the relative survival probability. Within this context and in case $x=0$ represents the absence of disease, the relative survival probability can be interpreted as the observed survival probability of the population studied, divided by the expected survival probability if the population was free of the disease of interest. Using (3), we can confirm the relative survival interpretation of our additive hazard model:

4 $$\begin{aligned} {\frac{S_{x}(t)}{S_{0, \theta }(t)} = \frac{\frac{S_{0, \theta }(t)}{\exp (t x\beta )}}{S_{0, \theta }(t)} = \frac{1}{\exp (t x\beta )}}. \end{aligned}$$

It should be noted that this relative survival interpretation is independent of the baseline distribution.

The likelihood function for the model can be derived by accounting for the fact that observations with an event contribute the logarithm of the pdf, and censored observations the logarithm of the survival function [7]. The contribution of a single observation *i* ($i = 1,...,N$) with covariate vector $x_i$ and observation time $t_i$ to the log-likelihood function $\ell _i$ is therefore

$$\begin{aligned} \ell _i&= (1-\delta _i)\log (f_{x_i}(t_i)) + \delta _i \log (S_{x_i}(t_i))\\&= (1-\delta _i) \log \left( \frac{f_0(t_i) + x_i\beta S_{0, \theta }(t)(t_i)}{\exp (t_ix_i\beta )} \right) + \delta _i \log \left( \frac{S_{0, \theta }(t)(t_i)}{\exp (t_ix_i\beta )} \right) \\&= (1-\delta _i)(\log (f_0(t_i)+x_i\beta S_{0, \theta }(t)(t_i))-t_ix_i\beta ) + \delta _i (\log (S_{0, \theta }(t)(t_i)) - t_ix_i\beta ). \end{aligned}$$

$\delta _i$ is the censoring indicator with $\delta _i=1$ if an observation is censored, and $\delta _i=0$ if an event has been observed. Parameter estimation is straightforward by maximizing the log-likelihood function with respect to the unknown regression coefficients $\beta$ and the parameters of the assumed baseline distribution. Each software that allows coding and maximizing such function, as for example SAS via the NLMIXED procedure or R via the optim-function, can be used to this task.

For practical application, it is possible to assume a wide range of baseline distributions, including for example the Exponential, Weibull, Gamma, Gompertz, Log-Normal and Log-Logistic distribution. For instance, assuming the Weibull distribution as baseline distribution and including a covariate $x_i$, the cdf is then given by:

5 $$\begin{aligned} F_{x_i}(t_i) = 1 - S_{x_i}(t_i) = 1 - \exp \left( -\left( \frac{t_i}{b_{WB}}\right) ^{a_{WB}} - t_i x_i\beta \right) . \end{aligned}$$

Alternatively, using a Log-Logistic distribution as baseline distribution, this leads to

6 $$\begin{aligned} F_{x_i}(t_i) = 1 - S_{x_i}(t_i) = 1 - \frac{\left( \left( \frac{t_i}{a_{LL}} \right) ^{b_{LL}} + 1 \right) ^{-1}}{\exp (t_i x_i \beta )}. \end{aligned}$$

$a_{WB}, b_{WB}, a_{LL}$ and $b_{LL}$ denote the distribution-specific parameters.

Results from applying the different baseline distributions can be compared via model selection criteria as the AIC or BIC. Parameters of main interest are finally the estimated regression coefficients and suitable transformations of the distribution parameters that have more intuitive interpretations, as the baseline mean or median of the assumed distribution.

### Simulation study

To evaluate the parametric additive hazard model, we conducted a simulation study. For comparison, we included the semi-parametric additive hazard model of Lin et al. [17]. The simulation study was implemented using R (version 4.1.2), the full code is publicly available on Zenodo [26].

#### Setting

Our parameter settings were motivated by the Halle Lung Carcinoma (HALLUCA) study which we also use as exemplary application in the Example section (Example: the HALLUCA study) [3]. The data from the HALLUCA study has been used in previous work proposing an extension of relative survival models for clustered responses [16] and are also suitable for our purpose. Accordingly, we focused on simulating a single binary covariate which can be interpreted for example as exposure in an observational study. Survival time is measured beginning with the day of diagnosis of lung cancer. As true underlying models used for data generation we assumed (*i*) the Weibull additive hazard model and (*ii*) the Log-Logistic additive hazard model as shown in equation (5) and (6). The true parameters of the distributions equaled their estimates from the HALLUCA study

- with $a_{WB}=0.86, b_{WB}=1.77$ for the Weibull distribution, and
- with $a_{LL}=1.06, b_{LL}=1.14$ for the Log-Logistic distribution.

In addition, we varied the number of participants per study which were set to 50 or 200. Moreover, we distinguish between a smaller number of observed events per study of 60% (40% censoring) and a higher number of 80% observed events (20% censoring). Further, we evaluated the true effect of the binary covariate for $\beta =0$, $\beta =0.8$ and $\beta =1.6$.

#### Data generation

Combining all parameters led to 24 different settings, i.e., 12 settings for each of (*i*) and (*ii*), for which data were generated. For every setting, we simulated 1000 data sets. Participants of each study were randomly allocated into two groups, as indicated by the binary covariate to which the finally estimated regression coefficient $\beta$ corresponds. We assumed that our two groups are of equal size. Survival times for each participant were generated from the true underlying distribution using inverse transform sampling. In case the covariate takes the value of zero, the cdf $F_{x_i}(t_i)$ from which we had to sample simply equals the Weibull or Log-Logistic distribution. Contrarily, if the covariate takes a value of one, the procedure is less trivial. In that case, it is not possible to invert the cdf analytically and we used numerical inversion to obtain random numbers from the respective function [5]. The likelihood of whether a study participant experienced an event or if the event is censored was generated from a Bernoulli distribution with respective success probability. To guarantee uninformative censoring, we multiplied the original survival time with a uniformly distributed random number in case the observation was censored.

#### Estimation methods and outcomes

For parameter estimation we used the Weibull and Log-Logistic additive hazard model. The corresponding likelihood functions were manually coded and implemented in the statistical software R. Optimization was done using the optim-function. True parameter values served as starting values for this procedure. As described above, we also applied the semi-parametric additive hazard model of Lin et al. [17] as comparative model. For this aim, we used the R-package addhazard [15] where the Lin and Ying model is already implemented. Outcome of primary interest was the estimated regression coefficient $\beta$ as this parameter is estimated by the parametric as well as by the semi-parametric model. The estimated parameters were compared in terms of bias, mean squared error (MSE) and empirical coverage. Moreover, we identified the number of converged simulation runs to assess numerical robustness.

## Results

### Simulation study

In reporting the results of the simulation study, we confine ourselves to the outcome of main interest, the estimated regression coefficient $\beta$. In the following, we give a brief overview of the results concerning bias, MSE, empirical coverage and numerical robustness. The complete simulation results can be found in Appendix II to V of the Additional file 1.

#### Bias

**Fig. 1:**
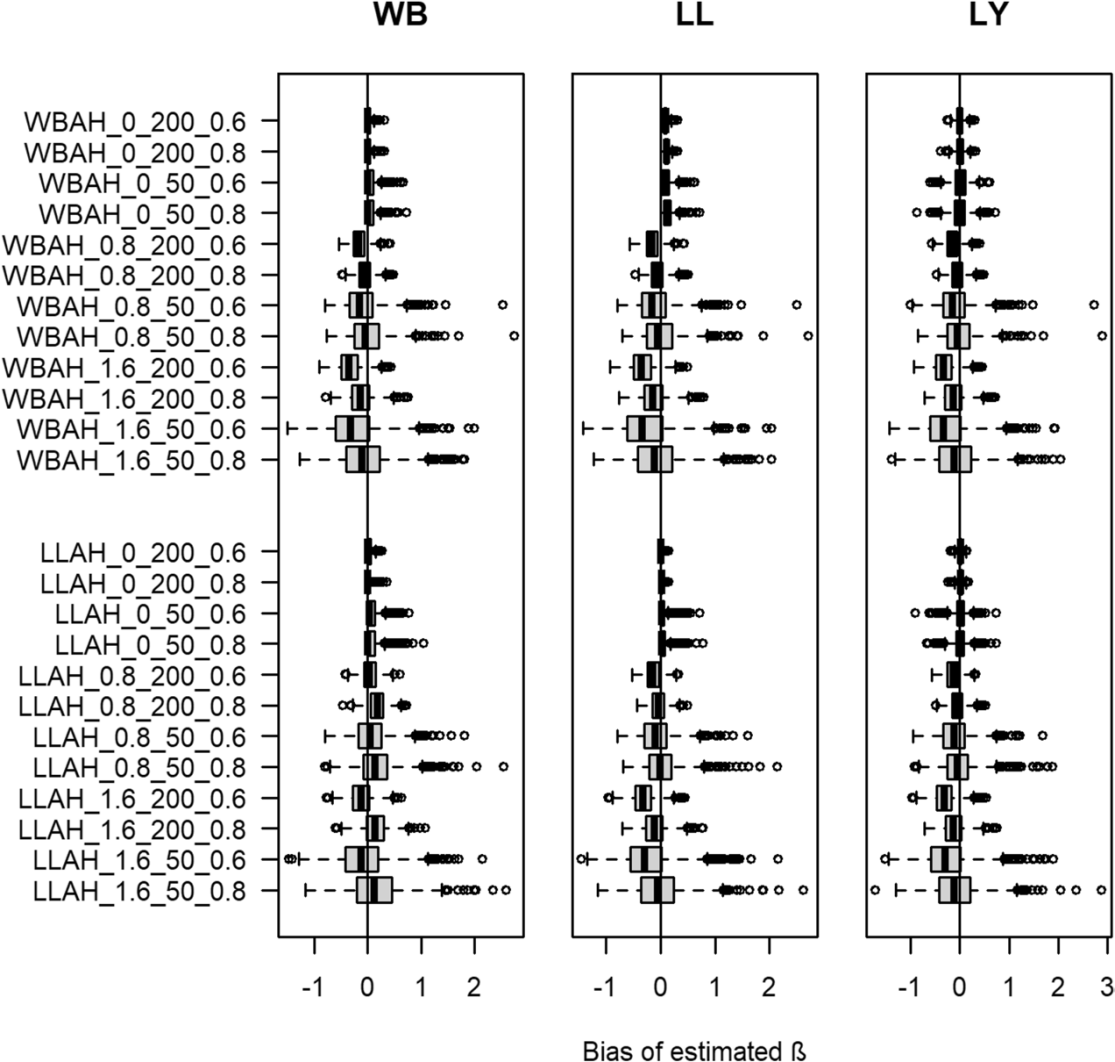
Box plots of estimated median bias for the regression coefficient $\beta$ over 1000 simulations for each setting. Y-axis denotes each setting with an ID consisting of the abbreviated true model (WBAH = Weibull additive hazard, LLAH = Log-Logistic additive hazard), the true $\beta$, the number of participants per study and number of events (e.g., Weibull additive hazard model, true $\beta = 0$, number of patients $= 50$, number of events = $60\%$ results in “WBAH_0_50_0.6”). Left-most plot shows results for the Weibull (WB) additive hazard model, middle plot shows results for the Log-Logistic (LL) additive hazard model and right plot shows results for the Lin-Ying (LY) model

Appendix II of the Additional file 1 provides the numerical results in terms of bias. Figure 1 illustrates the corresponding estimates. If the estimated additive hazard model is consistent with the true underlying distribution, we observe the best performance, i.e., smallest median bias, with some exceptions. Further, also considering that the estimated additive hazard model is consistent with the true underlying distribution, the additive hazard model slightly outperforms the semi-parametric model by Lin and Ying. This holds true for all estimated models, leading to a bias between $-0.36$ and 0.17. For most settings, the treatment effect is slightly underestimated which leads to a negative bias. The observed underestimation also holds for the Lin-Ying model which results in underestimation in more than 65% of all cases. We observe a change in the sign of the bias for settings where the Weibull-model is estimated using the new additive hazard model and relying on data that was generated via a Log-Logistic distribution. This underlines that in these settings the Weibull distribution is not an appropriate choice for modeling data that follows a Log-Logistic distribution, and an alternative choice of the underlying distribution should be made. The most extreme positive bias of 0.17 is observed for the estimated Weibull model when the Log-Logistic distribution, a true $\beta$ of 0.8, 20% censoring and 200 patients were assumed for data generation. The most extreme negative bias of $-0.35$ is observed for the estimated Weibull and Log-Logistic model in the setting with data generated from the Weibull additive hazard model, a true $\beta$ of 1.6, 40% censoring and 200 observations per sample. Generally, when data was generated assuming a Weibull distribution, all three models perform similarly. Though, if a Log-Logistic distribution is assumed as true, the estimated Log-Logistic and Lin-Ying model perform similarly, while results from the Weibull model slightly differ. The number of participants modeled per study minorly influences the median bias. The variability of the bias is smaller for higher numbers of observations (200 versus 50). Furthermore, the estimated models are sensitive towards the true value of $\beta$, where generally the bias is closer to zero and its variability is reduced for smaller values of $\beta$. In most cases, the bias is smaller in settings with more events (80% compared to 60%). With regards to the Weibull model, this only holds true if the estimated and true distribution are consistent.

#### MSE

**Fig. 2:**
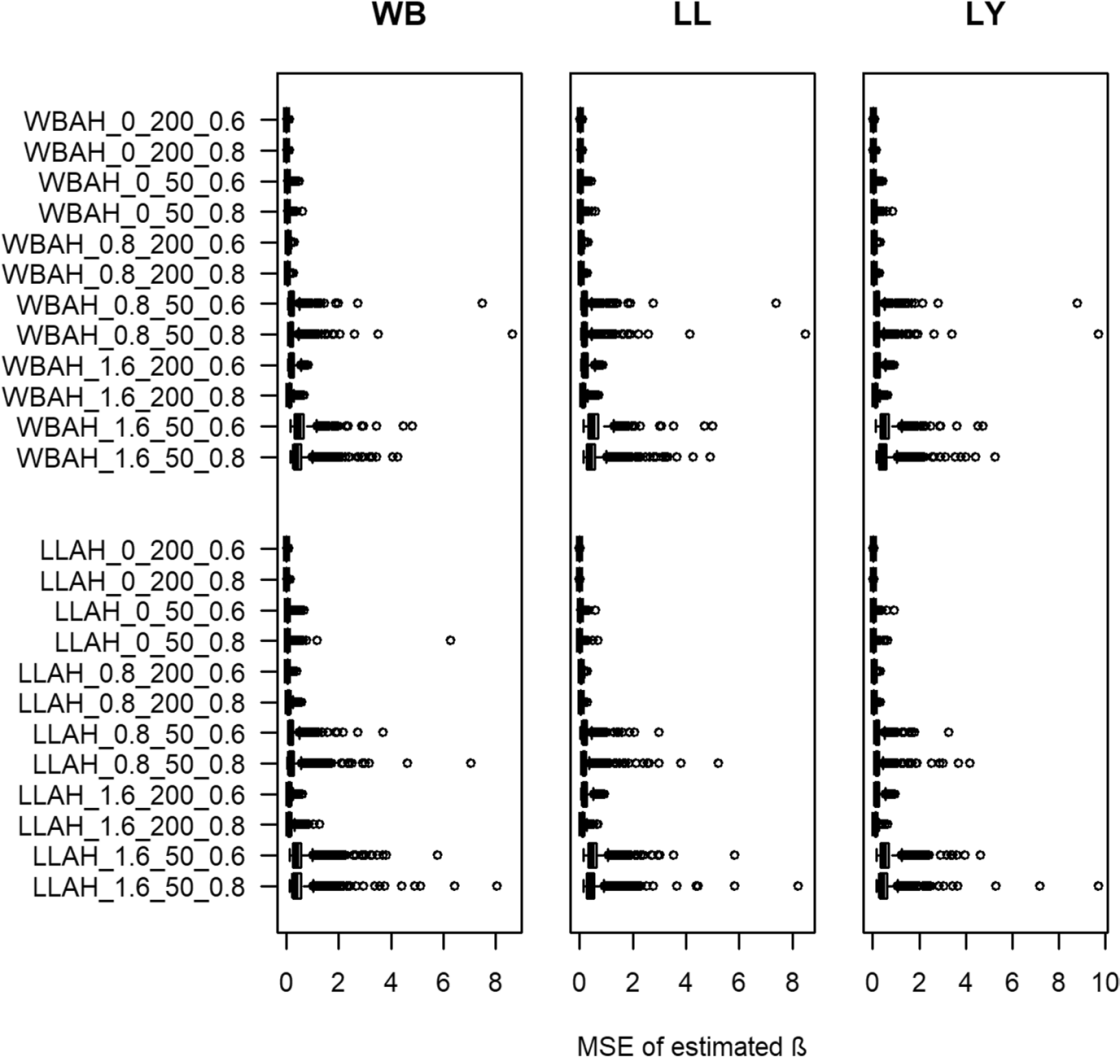
Box plots of estimated median mean squared error (MSE) for the regression coefficient $\beta$ over 1000 simulations for each setting. Y-axis denotes each setting with an ID consisting of the abbreviated true model (WBAH = Weibull additive hazard, LLAH = Log-Logistic additive hazard), the true $\beta$, the number of participants per study and number of events (e.g., Weibull additive hazard model, true $\beta = 0$, number of patients $= 50$, number of events = $60\%$ results in “WBAH_0_50_0.6”). Left-most plot shows results for the Weibull (WB) additive hazard model, middle plot shows results for the Log-Logistic (LL) additive hazard model and right plot shows results for the Lin-Ying (LY) model

Appendix III of the Additional file 1 shows the detailed results with regards to the MSE. Figure 2 visualizes the findings. Generally, there are hardly any differences for the MSE across the three estimated models for the respective settings. In some settings we observe an MSE of 0.00. These results indicate that the model works very well at minimum in these contexts. Contrarily, we observe a maximum error of 0.45 for the Lin-Ying model in one setting (true model based on Log-Logistic distribution, true $\beta$ = 1.6, number of observations = 50, number of events 60%). As for the bias, in most cases the MSE is slightly smaller when the estimated model is consistent with the true underlying distribution. For almost any setting, it holds true that the median MSE of the Weibull and Log-Logistic model is better than the MSE of the respective Lin-Ying model. The MSE seems to be negatively correlated with the number of participants per study, as for settings with 200 observations per sample (versus 50 observations) the MSE is smaller. Similar results are found with regards to the assumed number of events: the MSE decreases when more events (80% compared to 60%) are observed. Furthermore, for larger values of $\beta$, the MSE and its variability increase for each of the estimated models.

#### Empirical coverage

**Fig. 3:**
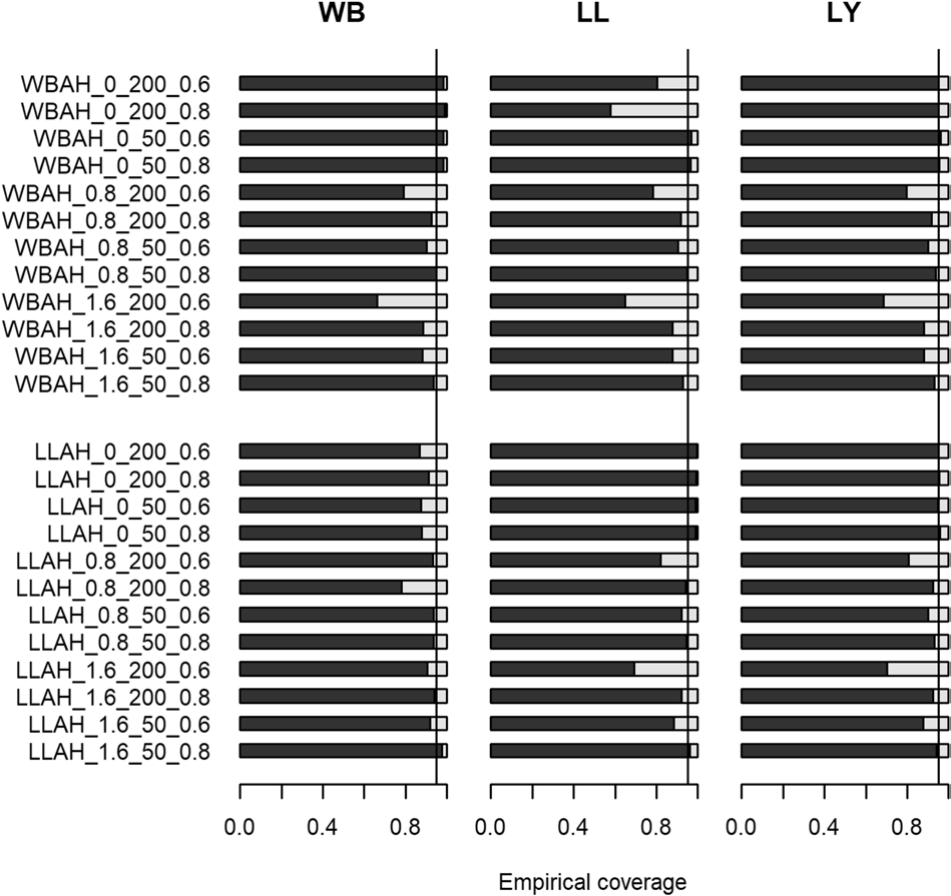
Bar plot showing relative frequency of coverage for the regression coefficient $\beta$ over 1000 simulations for each setting. Y-axis denotes each setting with an ID consisting of the abbreviated true model (WBAH = Weibull additive hazard, LLAH = Log-Logistic additive hazard), the true $\beta$, the number of participants per study and number of events (e.g., Weibull additive hazard model, true $\beta = 0$, number of patients $= 50$, number of events = $60\%$ results in “WBAH_0_50_0.6”). Left-most plot shows results for the Weibull (WB) additive hazard model, middle plot shows results for the Log-Logistic (LL) additive hazard model and right plot shows results for the Lin-Ying (LY) model

Appendix IV of the Additional file 1 provides detailed results about the models’ empirical coverage. Figure 3 illustrates the corresponding estimates. In terms of empirical coverage (on the 95% level), the performances of the three estimated models are relatively similar with some exceptions for a handful of settings. For the majority of the settings, the models again perform best when the estimated model and the true underlying distribution are equal. If the distributions are consistent, the Weibull and Log-Logistic model mostly outperform the Lin-Ying model, respectively. Overall, the empirical coverage often falls below 95%. Precisely, this is the case for 75% of the settings for the Weibull, for 71% of settings for the Log-Logistic and for 71% of all settings for the Lin-Ying model. The highest coverage of 100% is observed for the Log-Logistic model when data was generated from a Log-Logistic distribution with $\beta$ equal to 0, 60% events and 200 observations. On the other hand, the smallest coverage of 57.7% is observed for the Log-Logistic for data generated from a Weibull distribution with $\beta$ equal to 0, 80% events and 200 observations. Similar to bias and MSE, results depend on the true value of $\beta$. Coverage results for the estimated models seem to improve when $\beta$ is closer to zero. With regards to the number of events, results correlate positively with the event probability. Precisely, coverage results improve in settings where the event probability is higher (80% versus 60%). The number of participants per sample seems to have a slight influence on coverage results. However, the correlation of coverage and sample size is less clear for all three models compared to the correlation of the bias and study size.

#### Numerical robustness

Appendix V of the Additional file 1 shows the results concerning the models’ convergence. The numerical robustness is very satisfactory for all estimated time-to-event models. The Lin-Ying model performs most stable in terms of numerical robustness, always returning 1000 converged simulation runs. With respect to the Weibull model, the results are also very satisfactory. Only for two settings where data was generated from a Log-Logistic distribution, the model converges for 999 instead of 1000 simulation runs. The Log-Logistic model performs slightly less numerically robust with a minimum of 983 converged runs. In 75% of the settings, the Log-Logistic model achieves convergence for all 1000 generated data sets.

### Example: the HALLUCA study

For illustration purposes, we used data from the HALLUCA study, an epidemiological study that investigated medical care for lung cancer patients in the region of Halle (Saale) in the eastern part of Germany [3, 26]. In the HALLUCA study, a total of 1696 lung cancer patients were observed between April 1996 and September 2000. 1349 patients (79.5%) died until the end of follow-up. The median survival time in the population was 284 days (0.78 years). For the analysis reported here, we focused on a dichotomized version of the TNM-scale for the classification of malignant tumors as a predictor, where 739 patients had a TNM-scale of IIIb or smaller (TNM < IV), and 621 patients had a TNM-scale of IV (TNM IV). The 336 remaining patients for whom the TNM-scale was not reported, were deleted. The starting point for the survival definition relates to the day of diagnosis of lung cancer, while the end point of a patient’s survival time relates to death or the study end.

**Fig. 4:**
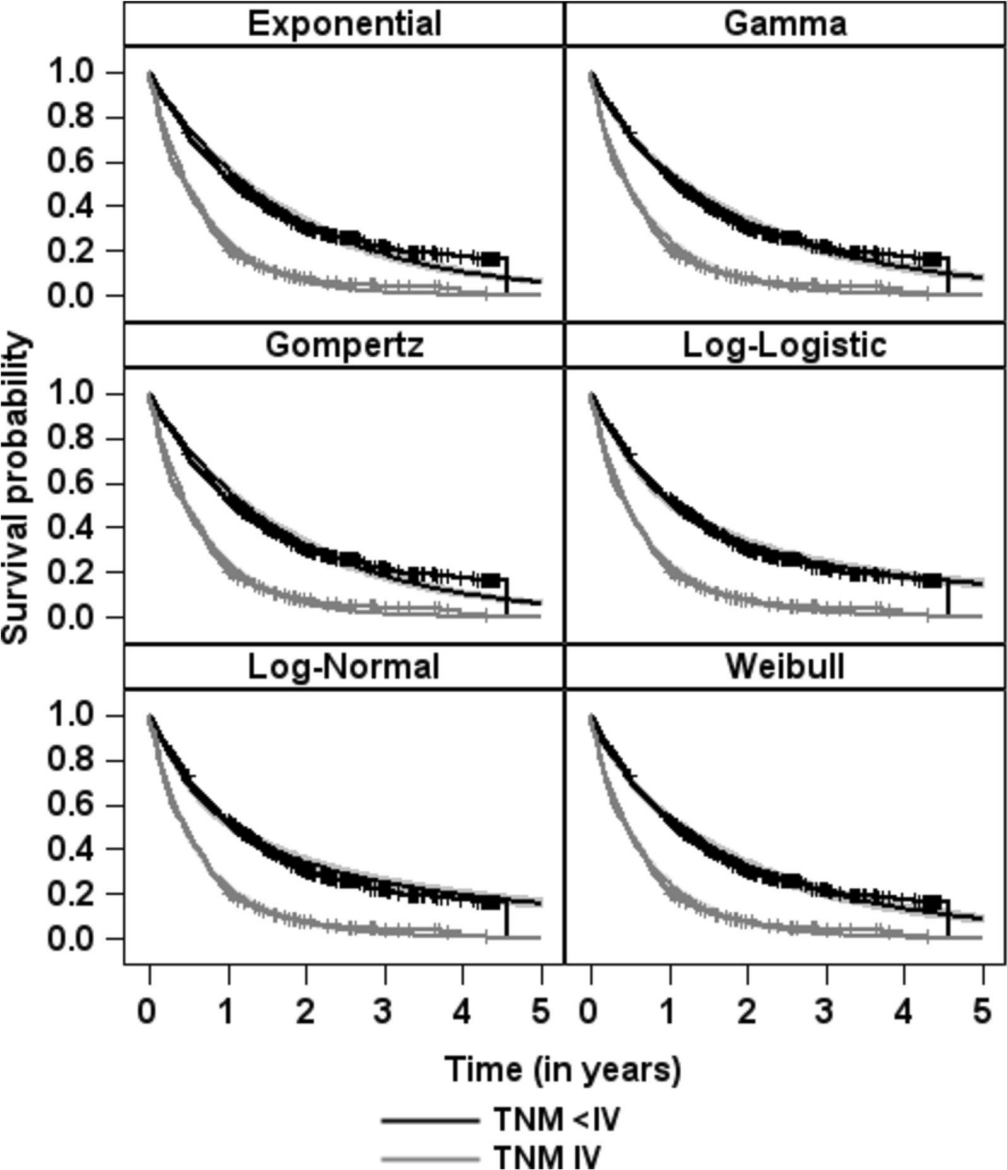
Estimated survival probabilities from the parametric additive hazard model using different distributions compared to the Kaplan-Meier curves

The Kaplan-Meier estimates for the two TNM classes and the estimated survival probabilities using the parametric additive hazard model with various distributions are given in Fig. 4. For the reference group (TNM < IV), for which we can estimate the parametric baseline distribution, the observed mean survival time is 1.69 years (95% confidence interval [1.56; 1.81]). The observed median survival time is 1.10 years [0.96; 1.24]. The figure shows that the estimated survival curves from the additive hazard model fit well with the Kaplan-Meier curves. For an increasing time *t* there are greater differences between the curves, especially for the Exponential, Weibull, Gamma and Gompertz model. However, for the Log-Normal and Log-Logistic distribution the fit is also quite well for larger survival times.

In Table 1, the results of the additive hazard model for six different baseline distributions are given. All models include a single binary covariate TNMIV, with (TNM < IV) being the reference group. Thus, we model the additive hazard of being in TNM stage IV. For evaluation purposes, we report the baseline mean and baseline median which can be compared to the empirical mean and median reported in the original HALLUCA study. To compare the model fit, we also report the Bayesian information criterion (BIC), where a smaller value indicates a better fit. The estimated regression coefficients for TNMIV show that being in stage IV increases the hazard by approximately 0.8. Being in an additive setting, the estimated $\beta$-value is added to the baseline distribution. That means being in the worse tumour group, the risk of dying rises because our model describes by how much the risk of dying changes in additive manner, not multiplicative. Alternatively, the coefficient could be interpreted in absolute terms, saying that when observing 100 people for one year, there are an additional 80 deaths among cancer patients in TNM class 4 compared to those with a lower TNM class. The results change slightly depending on the different distributions. Overall however, estimates from the parametric models are similar. In terms of the BIC, the Log-Logistic, Weibull and Gamma model fit best, returning almost identical parameter estimates and confidence intervals for $\beta _{\text {TNMIV}}$. With respect to the baseline location measures, the differences between the distributions are rather large, ranging from 1.77 to 7.90 for the baseline mean, and from 1.06 to 1.22 for the baseline median. For example, for the Log-Logistic approach, the model with the best BIC, we get a large baseline mean of 7.90 with a broad 95%-confidence interval of [3.39; 12.40]. With reference to Fig. 4, this can be explained by the flat survival curves at the end of the prediction range. Therefore, the baseline mean is necessarily larger with a broader confidence interval. However, regarding the baseline median, the Log-Logistic model yields an estimate of 1.06 which is in line with the empirical median of 1.10 from the Kaplan-Meier approach. The same holds true for the Log-Normal and Weibull model which achieve estimates that are also close to the empirical median.

**Table 1:** Estimates for the HALLUCA data set with 95% confidence intervals

Distribution	$\varvec{\beta }_{\textbf{TNMIV}}$	Baseline Mean	Baseline Median	No. of distribution parameters	BIC
Exponential	0.84 [0.72; 0.97]	1.77 [1.62; 1.92]	1.22 [1.12; 1.33]	1	2441.9
Weibull	0.78 [0.65; 0.91]	1.91 [1.70; 2.11]	1.15 [1.03; 1.26]	2	2427.1
Gamma	0.79 [0.67; 0.92]	1.85 [1.66; 2.03]	–	2	2431.8
Gompertz	0.84 [0.72; 0.97]	–	1.22 [1.12; 1.33]	2	2449.3
Log-Normal	0.80 [0.68; 0.92]	3.44 [2.75; 4.13]	1.07 [0.95; 1.19]	2	2450.3
Log-Logistic	0.78 [0.65; 0.91]	7.90 [3.39; 12.40]	1.06 [0.94; 1.18]	2	2417.6

**Fig. 5:**
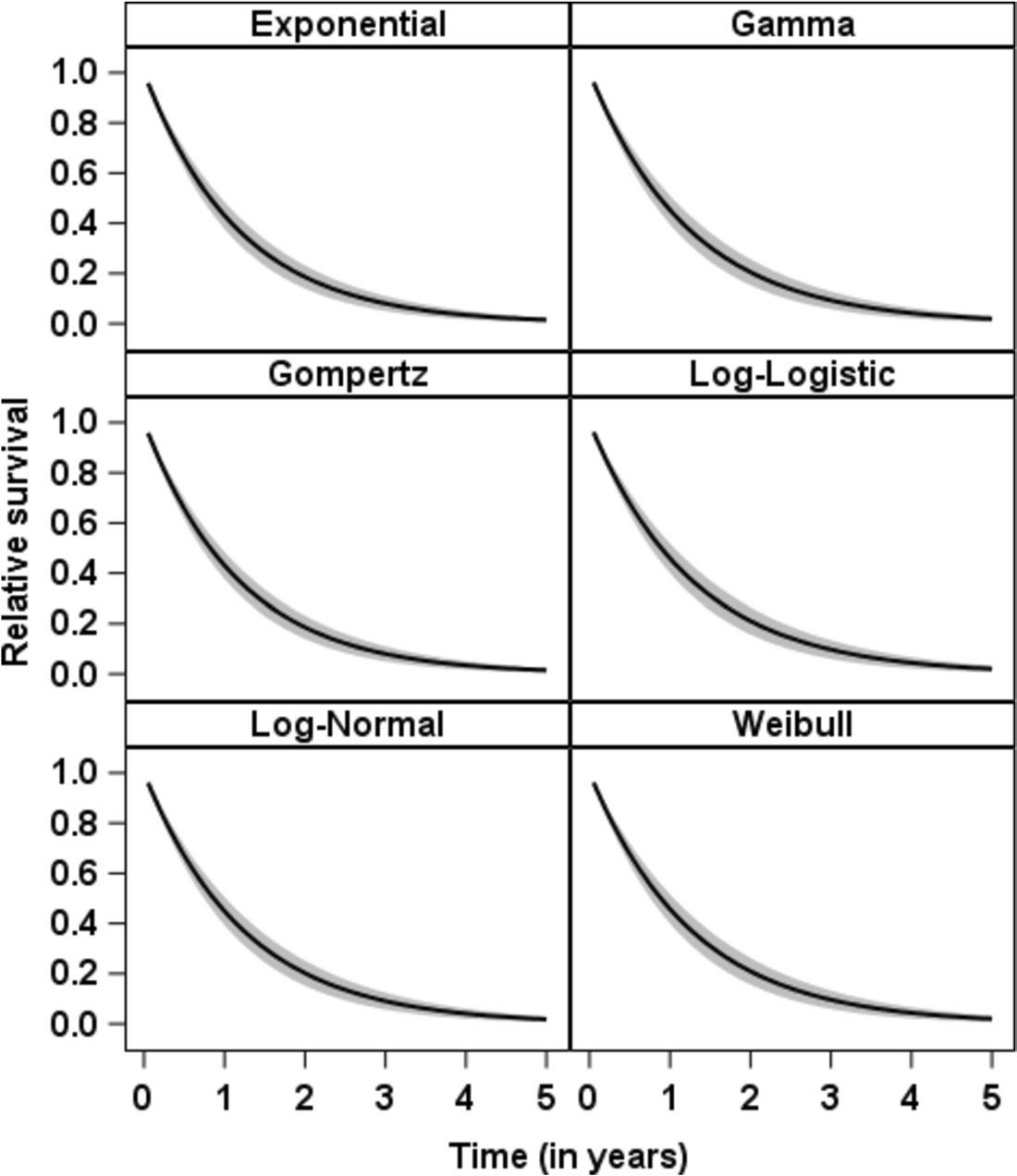
Relative survival probabilities from the parametric additive hazard model using different distributions. The gray shaded area depicts estimated confidence intervals

Figure 5 compares the relative survival probability from the parametric additive hazard model assuming different baseline distributions. The relative survival curves, taken as the ratio of survival in a group of individuals with TNM stage IV in comparison to the survival of a corresponding population with TNM < IV (see (4)), are rather similar between the various baseline distributions. It can easily be seen that survival strictly decreases over time, with a relative survival probability almost equal to zero after five years.

## Discussion

In this article, we propose a new parametric additive hazard model for time-to-event outcomes. Generally, parametric survival models are advisable as they are simpler, more informative and robust than non- or semi-parametric models [8, 10, 20]. In addition, they have hazard, survival and probability density functions directly available. We showed that the model is valid from a theoretical point of view. Further, in a simulation study as well as for an example from lung cancer research, we demonstrated that the model showed that the model works in practice. Convincingly, our proposed model can be implemented using any standard software that allows coding and maximizing a likelihood function. The corresponding SAS and R codes of our applications are publicly available on Zenodo [26].

The facilitated interpretation of our model is one of its most convincing advantages and is important for example from a clinical point of view. For instance, allowing to communicate outcomes of the additive hazard model in relative and absolute terms increases the comprehensibility of its results. Moreover, including time-independent (constant) covariates, our approach is advantageous over the additive hazard model of Aalen, since there, model coefficients often are difficult to understand as they change repeatedly over time and cannot be summarised easily. Furthermore, by modeling regression coefficients and distribution parameters that have more intuitive interpretations, our approach overcomes interpretational, mathematical and technical problems that arise when estimating hazard ratios as generic effect estimates. For practical application, several different distributions such as the Exponential, Weibull, Gamma, Gompertz, Log-Normal and Log-Logistic distribution can be implemented and compared via model selection criteria as the AIC or BIC. Moreover, parameter estimation of our model by the maximum likelihood principle is straightforward and can be accomplished with standard statistical software that allows for maximizing a likelihood function. Additionally, the proposed model is highly flexible in terms of possible extensions such as modeling correlated data by including random effects in the linear predictor, modeling non-linear covariate effects by splines, or by specifying baseline distributions that have more than two parameters. Maximum flexibility with respect to the baseline distribution can be obtained by using a piecewise-constant model, i.e., by dividing the observation period a-priori into intervals and assuming an exponential distribution in each of these intervals.

Evidently, there are some limitations with regards to our simulation study. The simulation was motivated by a real-world data example and thus offers a realistic setting, which we varied in a certain range of potential scenarios. However, these scenarios consider relatively small sample sizes and also rather moderate number of events. Consequently, it is not unlikely that the results of all models investigated in the simulation are biased in a certain extent and no optimal solution was found. For practical application, it should be considered positively that the treatment effect is more likely to be underestimated (versus overestimated). Thus, slightly biased inference in practical research may be less problematic than in a case of overestimation. Relying on the asymptotical properties and a correct implemented maximum likelihood estimation, we are confident that with an increased number of events and larger sample sizes, i.e., larger number of participants per study, the bias and its variability would asymptotically approach 0. Further, in practical research, it would evidently be an option to tune the parameters, settings and starting values of software functions used in the estimation to increase robustness and ensure convergence. For our study, we refrained from a comparison to the Cox model and instead used the Lin-Ying model for model evaluation. This is due to the fact that (*i*) the Lin-Ying model also relies on the additive hazards assumption and is thus a more direct competitor and (*ii*) our proposed model and the Cox model are inherently different and in a certain way incomparable. For instance, converting the additive into the multiplicative effect estimate is impossible. Further, the Cox model assumes multiplicative effects. Therefore, if data are generated using an additive model, it is obvious that the additive model will perform superior to the Cox model in terms of the effect estimator and its corresponding confidence interval. Vice versa, when generating data using the Cox model, we presume that our proposed parametric model would perform worse as it relies on the additivity assumption. However, it is left open for future research to assess whether p-values are consistent for the different models, even if they are simulated from the “wrong” model. Overall, the choice of using the Cox model or generally any additive model is dependent on the primary effect measure and study question. If the hazard ratio is of interest, then the Cox model would be preferable. If that is not the case, an additive model might be the better choice as it offers more easily interpretable effect measures. Further, the choice must be made under consideration of whether the user wants to assume multiplicativity or additivity. There is a number of methods for checking the assumptions and model fit for the Cox model, however, not all can be extended to the additive model [6]. For instance, we are not aware of any methods to confirm the validity of the additivity assumption or of the multiplicativity assumption, which would be the pendant specified in the Cox model. Although the direct comparison with a Cox model is generally questionable and outside the scope of this article, it nevertheless may remain interesting in case of insufficient knowledge about the effect of the covariates and whether to choose an additive or multiplicative model. We are unaware of an “overall”, combining model that contains both, additive and multiplicative models as a special case, and that has an additional parameter which measures the type of relationship (i.e., additive, multiplicative or something in between). The development of such a framework or model may be an interesting and valuable topic for future research.

With regards to the weaknesses of the proposed additive hazard model, our method suffers from the drawbacks that limits parametric versus semi- or non-parametric models. Non-parametric approaches generally require fewer assumptions about the data. Therefore, these may prove better when the true distribution is unknown and/or cannot be easily approximated. Vice versa, parametric methods are inherently dependent on the distribution chosen for estimation and on the assumption that this distribution is correctly specified. Further, it must be noted that parametric procedures require starting values for the optimization which may sometimes be complicated or problematic to define in practice. In case a distribution can be confidently specified to the data, parametric models will usually be more informative than semi- or non-parametric approaches. However, if this is not the case and the assumed distribution is false, results and conclusions are likely to be biased. For practical application, we recommend that the choice of the baseline distribution should be based on evidence from literature and previous research. However, if this is not applicable, and selection criteria such as the BIC or AIC is used for a data-based decision, this must be acknowledged in the reporting of the study and should be critically discussed. In that context, users should be cautious with any interpretation and should be are aware that confidence intervals for instance are potentially estimated too narrow. Besides, and by definition, our model assumes that the covariates act in an additive way to the baseline hazard function. Apparently, this assumption may be doubtful in some applications and in that case, other models may be more appropriate.

With regards to the framework of phases of methodological research Heinze et al. [13], the proposed additive hazard model currently belongs to early stages of methodological development, i.e., phase I or II. The aim here was to introduce a new idea, as well as to demonstrate its validity and its potential to improve on existing methods. Into the bargain, we derived the new methodological idea while providing, logical reasoning and proofs of empirical evidence through a real-world data example and a simulation study in a (yet) relatively narrow but suitable target setting. Carefully planned method comparison studies that investigate the model in future works could advance the proposed additive hazard model to later stages of methodological development and would be of great value for future users and the scientific community as a whole. Therefore, there is a need for future studies that explore the empirical properties of our model in a wider range of problems, highlight its advantages and limitations, and possibly uncover previously unknown behavior (e.g., in simulations with wide range of scenarios and different outcome types and realistic or complex comparative example data analyses).

## Conclusion

To summarize, the proposed parametric additive hazard model can serve as a powerful tool to analyze time-to-event outcomes. By definition, it simplifies interpretation, facilitates parameter estimation and permits greater flexibility than most existing and commonly used methods.

### Supplementary Information

**Supplementary material 1.**

## Data Availability

The datasets generated and/or analysed during the current study are available in the Zenodo repository, https://doi.org/10.5281/zenodo.7124989 [26].
