# Supplementary material for: A parametric additive hazard model for time-to-event analysis
Source: BMC Med Res Methodol. 2024 Feb 24;24:48. doi: 10.1186/s12874-024-02180-y (PMC10893628; doi:10.1186/s12874-024-02180-y)
Supplement: Supplementary file 1 — Supplementary material 1. [file 12874_2024_2180_MOESM1_ESM.pdf]

Additional file 1 to: A parametric additive hazard model for time-to-event analysis

Dina Voeltz [1,2*◇], Annika Hoyer[1*], Amelie Forkel[2], Anke Schwandt[3], Oliver Kuss[4]

The following additional information are available as part of the online article:

(i) Appendix I - Derivation of the additive hazard model

(ii) Appendix II - Bias for the estimated regression coefficient β

(iii) Appendix III - Mean squared error (MSE) for the estimated regression coefficient β

(iv) Appendix IV - Empirical coverage (in %) for the estimated regression coefficient β

(v) Appendix V - Number of converged simulation runs per setting (maximum number: 1000)

(vi) Appendix VI - Box plots of estimated variance for the regression coefficient β

[1] Biostatistics and Medical Biometry, Medical School OWL, Bielefeld University, Bielefeld
[2] Department of Statistics, Ludwig-Maximilians-University Munich
[3] Nuvisan GmbH, Neu-Ulm
[4] German Diabetes Center, Leibniz Center for Diabetes Research at Heinrich Heine University Düsseldorf, Institute for Biometrics and Epidemiology
[*] These authors contributed equally to this work.
[◇] Correspondence to: Dina Voeltz, Biostatistics and Medical Biometry, Medical School OWL, Bielefeld University, Universitätsstr. 25, 33615 Bielefeld, Germany. E-Mail: dina.voeltz@uni-bielefeld.de

Appendix I: Derivation of the additive hazard model

$$
\begin{aligned}
f_x(t) &= \left(\frac{f_0(t)}{S_{0,\theta}(t)} + x\beta\right) S_x(t) \\
&= \left(\frac{f_0(t)}{S_{0,\theta}(t)} + x\beta\right) \exp(-H_x(t)) \\
&= \left(\frac{f_0(t)}{S_{0,\theta}(t)} + x\beta\right) \exp\left(-\int_0^t h_x(u)du\right) \\
&= \left(\frac{f_0(t)}{S_{0,\theta}(t)} + x\beta\right) \exp\left(-\int_0^t h_{0,\theta}(u) + x\beta du\right) \\
&= \left(\frac{f_0(t)}{S_{0,\theta}(t)} + x\beta\right) \exp\left(-\int_0^t h_{0,\theta}(u)du - \int_0^t x\beta du\right) \\
&= \left(\frac{f_0(t)}{S_{0,\theta}(t)} + x\beta\right) \frac{\exp\left(-\int_0^t h_{0,\theta}(u)du\right)}{\exp\left(\int_0^t x\beta du\right)} \\
&= \left(\frac{f_0(t)}{S_{0,\theta}(t)} + x\beta\right) \frac{S_{0,\theta}(t)}{\exp\left(\int_0^t x\beta du\right)} \\
&= \left(\frac{f_0(t)}{S_{0,\theta}(t)} + x\beta\right) \frac{S_{0,\theta}(t)}{\exp(tx\beta)} \\
&= \frac{f_0(t)}{\exp(tx\beta)} + \frac{x\beta S_{0,\theta}(t)}{\exp(tx\beta)} \\
&= \frac{f_0(t) + x\beta S_{0,\theta}(t)}{\exp(tx\beta)}
\end{aligned}
$$

Appendix II: Bias

Table 1: Results for the median bias (25% quartile [Q1]; 75% quartile [Q3]) of the estimated regression coefficient β

True model/ true β / nPers/ events	Estimated model: Weibull Q1	Weibull Median	Weibull Q3	Log-Logistic Q1	Log-Logistic Median	Log-Logistic Q3	Lin-Ying Q1	Lin-Ying Median	Lin-Ying Q3
**WBAH_0_200_0.6**	0.00	0.00	0.05	0.06	0.08	0.11	-0.05	0.00	0.05
**WBAH_0_200_0.8**	0.00	0.00	0.05	0.08	0.10	0.14	-0.06	0.00	0.05
**WBAH_0_50_0.6**	0.00	0.00	0.10	0.03	0.09	0.15	-0.09	0.01	0.10
**WBAH_0_50_0.8**	0.00	0.00	0.10	0.06	0.11	0.17	-0.11	0.00	0.10
**WBAH_0.8_200_0.6**	-0.26	-0.16	-0.06	-0.26	-0.16	-0.06	-0.26	-0.16	-0.06
**WBAH_0.8_200_0.8**	-0.16	-0.06	0.04	-0.16	-0.07	0.03	-0.16	-0.07	0.04
**WBAH_0.8_50_0.6**	-0.34	-0.16	0.10	-0.35	-0.16	0.09	-0.34	-0.16	0.09
**WBAH_0.8_50_0.8**	-0.25	-0.05	0.21	-0.26	-0.06	0.21	-0.26	-0.06	0.19
**WBAH_1.6_200_0.6**	-0.49	-0.35	-0.19	-0.50	-0.36	-0.19	-0.48	-0.34	-0.18
**WBAH_1.6_200_0.8**	-0.29	-0.14	0.02	-0.31	-0.15	0.02	-0.30	-0.14	0.02
**WBAH_1.6_50_0.6**	-0.59	-0.33	0.03	-0.61	-0.34	0.03	-0.61	-0.34	0.01
**WBAH_1.6_50_0.8**	-0.39	-0.10	0.22	-0.42	-0.12	0.21	-0.42	-0.12	0.22
**LLAH_0_200_0.6**	0.00	0.00	0.06	0.00	0.00	0.01	-0.02	0.00	0.03
**LLAH_0_200_0.8**	0.00	0.00	0.01	0.00	0.01	0.02	-0.02	0.00	0.04
**LLAH_0_50_0.6**	0.00	0.02	0.13	0.00	0.01	0.05	-0.06	0.01	0.08
**LLAH_0_50_0.8**	0.00	0.00	0.13	0.00	0.01	0.07	-0.08	0.00	0.07
**LLAH_0.8_200_0.6**	-0.06	0.04	0.15	-0.24	-0.14	-0.03	-0.25	-0.15	-0.04
**LLAH_0.8_200_0.8**	0.06	0.18	0.29	-0.15	-0.05	0.05	-0.16	-0.06	0.04
**LLAH_0.8_50_0.6**	-0.17	0.04	0.25	-0.31	-0.11	0.11	-0.34	-0.13	0.09
**LLAH_0.8_50_0.8**	-0.08	0.14	0.37	-0.22	-0.01	0.19	-0.25	-0.06	0.15
**LLAH_1.6_200_0.6**	-0.27	-0.12	0.03	-0.47	-0.33	-0.18	-0.47	-0.32	-0.17
**LLAH_1.6_200_0.8**	-0.02	0.14	0.30	-0.27	-0.13	0.03	-0.28	-0.13	0.03
**LLAH_1.6_50_0.6**	-0.42	-0.13	0.20	-0.56	-0.30	0.01	-0.58	-0.31	0.01
**LLAH_1.6_50_0.8**	-0.19	0.12	0.46	-0.36	-0.07	0.24	-0.42	-0.13	0.21

Appendix III: Mean squared error

Table 2: Results for the median mean squared error (MSE) (25% quartile [Q1]; 75% quartile [Q3]) of the estimated regression coefficient β

	Estimated model								
True model/	Weibull			Log-Logistic			Lin-Ying		
true β / nPers/ events	Q1	Median	Q3	Q1	Median	Q3	Q1	Median	Q3
**WBAH_0_200_0.6**	0.00	0.01	0.01	0.01	0.01	0.02	0.01	0.01	0.01
**WBAH_0_200_0.8**	0.01	0.01	0.01	0.01	0.01	0.02	0.01	0.01	0.01
**WBAH_0_50_0.6**	0.02	0.03	0.04	0.01	0.02	0.04	0.02	0.04	0.06
**WBAH_0_50_0.8**	0.02	0.03	0.04	0.01	0.03	0.05	0.03	0.04	0.07
**WBAH_0.8_200_0.6**	0.03	0.05	0.09	0.03	0.05	0.09	0.03	0.05	0.09
**WBAH_0.8_200_0.8**	0.03	0.04	0.06	0.03	0.04	0.06	0.03	0.04	0.06
**WBAH_0.8_50_0.6**	0.12	0.17	0.27	0.13	0.17	0.26	0.13	0.17	0.28
**WBAH_0.8_50_0.8**	0.11	0.16	0.26	0.12	0.16	0.25	0.12	0.17	0.26
**WBAH_1.6_200_0.6**	0.10	0.18	0.29	0.10	0.18	0.30	0.10	0.17	0.29
**WBAH_1.6_200_0.8**	0.07	0.09	0.16	0.07	0.10	0.16	0.07	0.10	0.17
**WBAH_1.6_50_0.6**	0.31	0.42	0.65	0.32	0.44	0.70	0.33	0.45	0.70
**WBAH_1.6_50_0.8**	0.27	0.35	0.56	0.27	0.37	0.57	0.28	0.38	0.59
**LLAH_0_200_0.6**	0.00	0.00	0.01	0.00	0.00	0.00	0.00	0.00	0.00
**LLAH_0_200_0.8**	0.00	0.01	0.01	0.00	0.00	0.00	0.00	0.00	0.00
**LLAH_0_50_0.6**	0.01	0.02	0.04	0.00	0.01	0.02	0.01	0.02	0.04
**LLAH_0_50_0.8**	0.02	0.03	0.05	0.00	0.00	0.02	0.01	0.02	0.04
**LLAH_0.8_200_0.6**	0.02	0.03	0.06	0.03	0.05	0.08	0.03	0.05	0.09
**LLAH_0.8_200_0.8**	0.03	0.06	0.11	0.03	0.04	0.06	0.03	0.04	0.06
**LLAH_0.8_50_0.6**	0.09	0.14	0.25	0.12	0.16	0.25	0.12	0.18	0.27
**LLAH_0.8_50_0.8**	0.10	0.15	0.28	0.11	0.15	0.22	0.12	0.16	0.25
**LLAH_1.6_200_0.6**	0.06	0.09	0.14	0.10	0.17	0.27	0.10	0.17	0.28
**LLAH_1.6_200_0.8**	0.06	0.08	0.15	0.07	0.09	0.14	0.07	0.10	0.15
**LLAH_1.6_50_0.6**	0.25	0.35	0.55	0.31	0.41	0.62	0.33	0.44	0.69
**LLAH_1.6_50_0.8**	0.23	0.32	0.55	0.27	0.34	0.53	0.29	0.38	0.60

Appendix IV: Empirical coverage

Table 3: Empirical coverage (in %) for the estimated regression coefficient β

True model/ true β/ nPers/ events	Estimated model		
	Weibull	Log-Logistic	Lin-Ying
	β	β	β
WBAH_0_200_0.6	98.1	80.3	95.1
WBAH_0_200_0.8	99.0	57.7	95.1
WBAH_0_50_0.6	98.2	97.0	96.0
WBAH_0_50_0.8	98.2	96.4	95.3
WBAH_0.8_200_0.6	78.9	78.2	79.5
WBAH_0.8_200_0.8	92.5	91.9	91.7
WBAH_0.8_50_0.6	90.3	90.3	90.1
WBAH_0.8_50_0.8	95.0	94.8	93.9
WBAH_1.6_200_0.6	66.4	64.9	68.5
WBAH_1.6_200_0.8	88.4	87.6	88.1
WBAH_1.6_50_0.6	88.0	87.6	88.0
WBAH_1.6_50_0.8	93.4	92.8	93.2
LLAH_0_200_0.6	86.9	100.0	95.2
LLAH_0_200_0.8	91.3	99.3	95.6
LLAH_0_50_0.6	87.3	98.7	94.7
LLAH_0_50_0.8	87.8	99.0	95.9
LLAH_0.8_200_0.6	93.1	81.9	80.7
LLAH_0.8_200_0.8	78.1	94.0	92.3
LLAH_0.8_50_0.6	93.3	92.2	90.1
LLAH_0.8_50_0.8	93.6	94.6	93.0
LLAH_1.6_200_0.6	90.6	69.2	70.3
LLAH_1.6_200_0.8	93.8	92.3	92.4
LLAH_1.6_50_0.6	91.7	88.6	87.6
LLAH_1.6_50_0.8	97.7	96.1	94.1

Appendix V: Number of converged simulation runs

Table 4: Number of converged simulation runs per setting (maximum number: 1000)

True model/ true β/	Estimated model		
nPers/ events	Weibull	Log-Logistic	Lin-Ying
WBAH_0_200_0.6	1000	1000	1000
WBAH_0_200_0.8	1000	1000	1000
WBAH_0_50_0.6	1000	999	1000
WBAH_0_50_0.8	1000	1000	1000
WBAH_0.8_200_0.6	1000	1000	1000
WBAH_0.8_200_0.8	1000	1000	1000
WBAH_0.8_50_0.6	1000	1000	1000
WBAH_0.8_50_0.8	1000	999	1000
WBAH_1.6_200_0.6	1000	1000	1000
WBAH_1.6_200_0.8	1000	1000	1000
WBAH_1.6_50_0.6	1000	1000	1000
WBAH_1.6_50_0.8	1000	1000	1000
LLAH_0_200_0.6	999	995	1000
LLAH_0_200_0.8	1000	983	1000
LLAH_0_50_0.6	1000	996	1000
LLAH_0_50_0.8	1000	990	1000
LLAH_0.8_200_0.6	1000	1000	1000
LLAH_0.8_200_0.8	1000	1000	1000
LLAH_0.8_50_0.6	999	1000	1000
LLAH_0.8_50_0.8	1000	1000	1000
LLAH_1.6_200_0.6	1000	1000	1000
LLAH_1.6_200_0.8	1000	1000	1000
LLAH_1.6_50_0.6	1000	1000	1000
LLAH_1.6_50_0.8	1000	1000	1000

Appendix VI - Box plots of estimated variance for the regression coefficient β

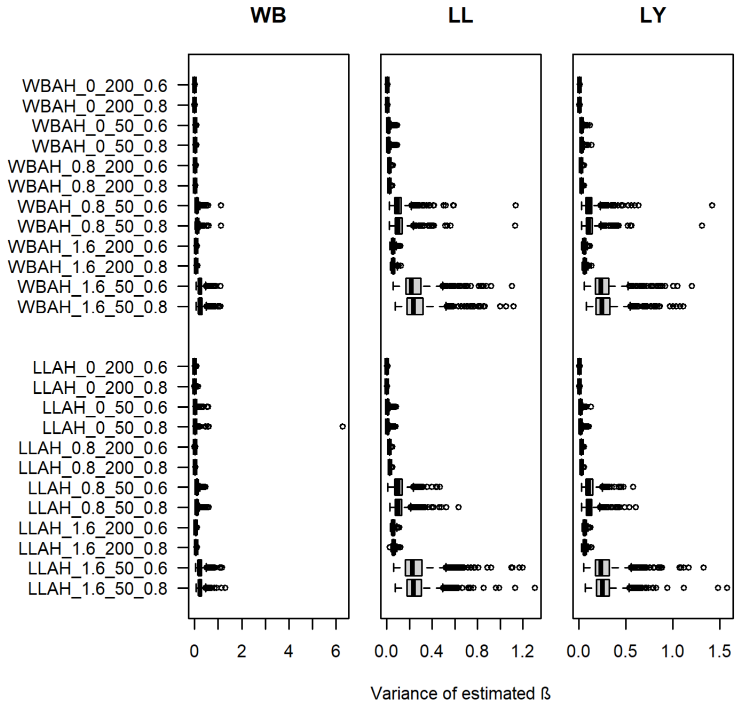

Figure 1: Box plots of estimated variance for the regression coefficient β over 1000 simulations for each setting. Y-axis denotes each setting with an ID consisting of the abbreviated true model (WBAH = Weibull additive hazard, LLAH = Log-Logistic additive hazard), the true β, the number of participants per study and number of events (e.g., Weibull additive hazard model, true $\beta = 0$, number of patients = 50, number of events = 60% results in "WBAH_0_50_0.6"). Left-most plot shows results for the Weibull (WB) additive hazard model, middle plot shows results for the Log-Logistic (LL) additive hazard model and right plot shows results for the Lin-Ying (LY) model.
